# Effect of SGLT2 inhibitors on the proteinuria reduction in patients with IgA nephropathy

**DOI:** 10.3389/fmed.2023.1242241

**Published:** 2023-09-06

**Authors:** Yaping Dong, Sufang Shi, Lijun Liu, Xujie Zhou, Jicheng Lv, Hong Zhang

**Affiliations:** ^1^Renal Division, Peking University First Hospital, Peking University Institute of Nephrology, Beijing, China; ^2^Key Laboratory of Renal Disease, Ministry of Health of China, Key Laboratory of Chronic Kidney Disease Prevention and Treatment (Peking University), Ministry of Education, Beijing, China; ^3^Research Units of Diagnosis and Treatment of Immune-Mediated Kidney Diseases, Chinese Academy of Medical Sciences, Beijing, China

**Keywords:** IgA nephropathy, sodium-glucose cotransporter 2 inhibitors, proteinuria, reninangiotensin-aldosterone system inhibitors, immunosuppressive agents

## Abstract

**Backgroud:**

Recent trials suggest sodium-glucose cotransporter 2 inhibitors (SGLT2i) significantly reduced proteinuria in patients with IgA nephropathy (IgAN). While little was known its efficacy in clinical practice especially in those already received full dose reninangiotensin-aldosterone system (RAAS) inhibitors.

**Methods:**

A cohort of 93 Chinese patients with biopsy-proven IgAN and persistent proteinuria underwent full supportive therapy, including optimal blood pressure control and full dose angiotensin-converting enzyme–inhibitor or angiotensin receptor blocker therapy. Proteinuria reduction at three and six months after initiating SGLT2i therapy was analyzed.

**Results:**

A total of 93 patients were enrolled in this study and 62 of them completed the six-month follow-up. After SGLT2i administration, a significant reduction in proteinuria was observed, with a decrease of 22.9% (*p* < 0.001) at three months and 27.1% (*p* < 0.001) at six months. During the six-month follow-up period, a decline of 3.0 mL/min/1.73m^2^ in estimated glomerular filtration rate (eGFR) (*p* = 0.012) and an increase of 0.8 g/L in albumin (*p* = 0.017) were observed. The anti-hypertensive effect of SGLT2i was not significant (*p* > 0.05). Notably, a consistent antiproteinuric effect of SGLT2i was observed across various settings, including different age groups, baseline levels of proteinuria/eGFR, use of immunosuppressive agents, and the presence of comorbid diabetes and hypertension (all *p* values >0.05).

**Conclusion:**

The proteinuria was significantly reduced after SGLT2i administration in IgAN patients with full dose angiotensin-converting enzyme–inhibitor or angiotensin receptor blocker therapy. Importantly, the antiproteinuric effect of SGLT2i was observed independently of immunosuppressive agent therapy, age, baseline eGFR and proteinuria levels, as well as the history of hypertension and diabetes.

## 1. Introduction

IgA nephropathy (IgAN) is the most prevalent primary glomerular disease worldwide, but the therapeutic strategy for IgAN is limited, with unsatisfactory treatment effects. According to the 2021 Kidney Diseases: Improving Global Outcomes (KDIGO) guideline, the optimized supportive treatments, such as blood control with renin-angiotensin-aldosterone system (RAAS) blockers, are the mainstay of IgAN management (1). Glucocorticoid administration may be considered as an intensive therapy who exhibit persistent proteinuria exceeding 0.75–1.0 g per day. While the side effects of glucocorticoid use, such as infection and metabolic disorder, are not negligible, especially for those with concomitant diabetes, obesity and osteoporosis (2). More effective treatment choices are required in this field.

Proteinuria is a prominent characteristic of IgA nephropathy (IgAN) and serves as a predictive factor for renal function decline and increased mortality when persistently exceeding 1 g/day (3). The reduction of proteinuria has become a crucial therapeutic purpose for patients with IgAN. Recent studies with compelling evidence suggested the protective effect of sodium-glucose cotransport 2 inhibitors (SGLT2i), the novel antidiabetic agents, on renal function (4–6). For example, empagliflozin reduced the risk of incident or worsening nephropathy by 39% in patients with diabetes. Moreover, SGLT2i prevents renal disease progression regardless of the plasm glucose level (7, 8). The proteinuria-decreasing effect of SGLT2i at least partly explains its clinical benefits. Based on the results from the DAPA-CKD trial, dapagliflozin attenuated albuminuria by 26% and significantly reduced the risk of major adverse kidney events by 71% in patients with IgAN compared to the placebo group (9). However, the participants from DAPA-CKD only received RAASi for four weeks, which is much shorter than the treatment course of three months recommended by the KDIGO guideline (10). Additionally, further studies are required to validate the potential of SGLT2 inhibitors in reducing proteinuria among patients with IgAN.

In the present study, we evaluate the effect of SGLT2i on proteinuria reduction in IgAN patients with persistent proteinuria in the Chinese IgAN population. A large prospective cohort of IgAN patients was applied for participant enrollment and data analysis.

## 2. Materials and methods

### 2.1. Study population

**Figure 1:**
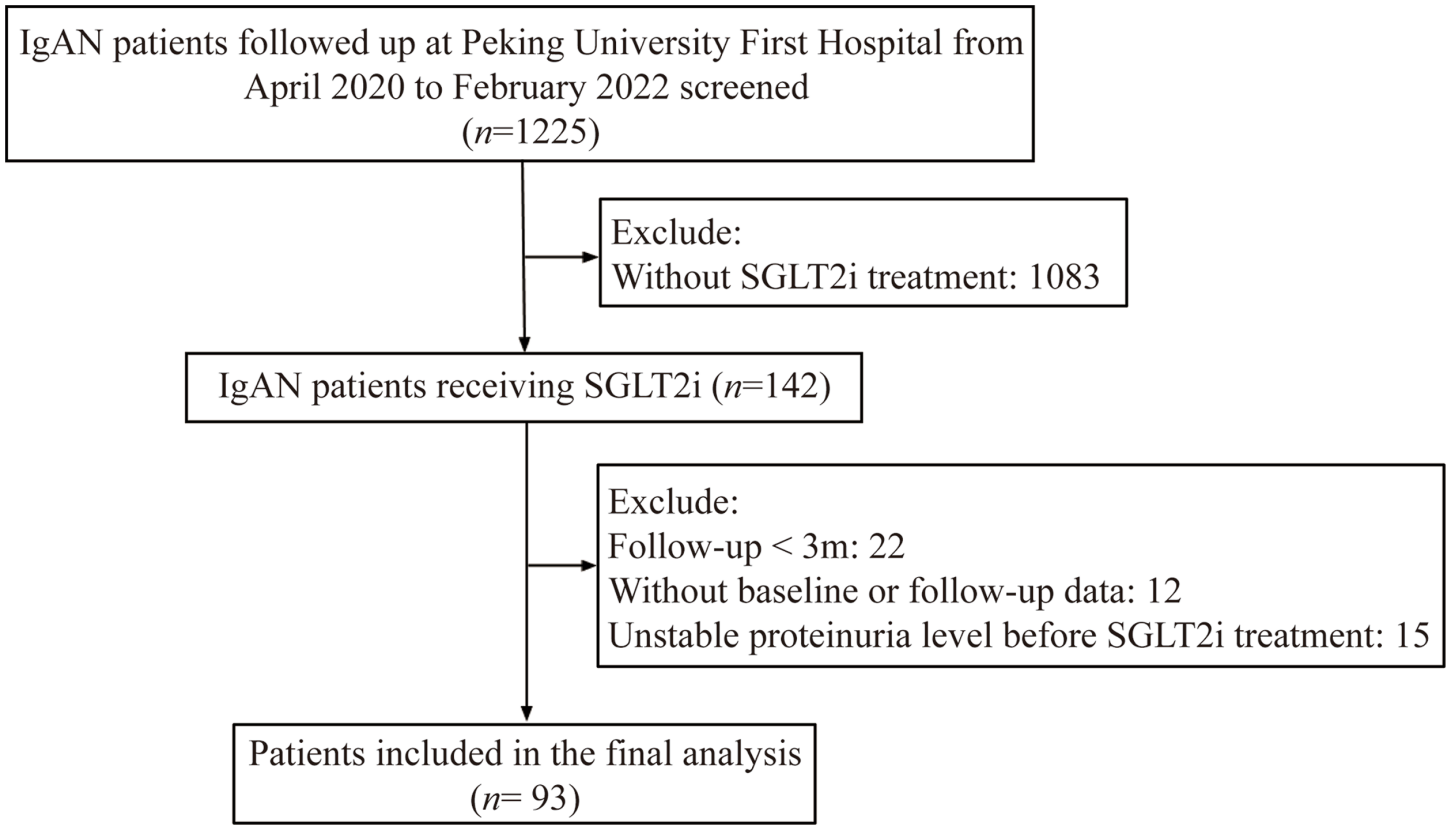
Flow chart of study population. IgAN: IgA nephropathy; SGLT2i: sodium-glucose cotransporter 2 inhibitors; Unstable proteinuria level indicates: (proteinuria of three months before SGLT2i use - baseline proteinuria) / baseline proteinuria >0.5.

From April 2020 to February 2022, a total of 1,225 patients with biopsy-proven IgAN were followed up at Peking University First Hospital. Among them, 142 patients received treatment with SGLT2i. Among them, 22 patients with less than three months of follow-up and 12 patients without regular proteinuria monitoring were excluded. Additionally, 15 patients with unstable proteinuria levels before SGLT2i treatment were also excluded from this study. Finally, the remaining 93 patients were included in our final analysis (shown in Figure 1). All patients had already received Full dose [maximum tolerable or labeled (whichever is reached first) dose] of either an ACE inhibitor or an ARB for at least three months but still had a baseline proteinuria >0.5 g/d. The study was approved by Peking University First Hospital. Written informed consent was obtained from all participants.

All participants were administered either dapagliflozin at a daily dose of 5 mg or 10 mg, or canagliflozin at a daily dose of 100 mg, for a minimum of three months. Follow-up visits were scheduled every 3 months. Blood and urine samples were collected at baseline and each visit for clinical laboratory measurements.

### 2.2. Data collection

Clinical data, including gender, age at the time of renal biopsy, and systolic/diastolic blood pressure, serum creatine, hematuria and 24-h urine protein excretion, were measured and recorded at the time of renal biopsy and each visit. Medication usage was collected during the follow-up period, including the administration of RAAS blockers, steroids, and other immunosuppressants. In our study, a resting systolic blood pressure (SBP) of ≥140 mmHg and/or diastolic blood pressure (DBP) of ≥90 mmHg, or a self-reported history of high blood pressure was defined as hypertension. The estimated glomerular filtration rate (eGFR) was calculated according to the Chronic Kidney Disease Epidemiology Collaboration (CKD-EPI) equation (11). The Oxford classification was used for the evaluation of pathological (12).

### 2.3. Statistical analysis

The continuous data were classified into normal and abnormal distribution *via* the normality test, which represented as mean ± standard deviation (SD) or median (interquartile range, IQR), respectively. Categorical data are summarized as percentages and statistically evaluated *via* Chi-square tests. The comparison between two groups were evaluated with SPSS 19.0 software (IBM Corporation, New York, United States). Paired t test was applied for the parametric statistic while Wilcoxon signed-rank test was performed for the non-parametric statistic. The trend in proteinuria reduction was assessed by one-way ANOVA with repeated measures. The slope of the linear regression curve was determined by calculating the rate of decrease in eGFR and proteinuria before and after treatment with an SGLT2 inhibitor. To avoid potential bias in our findings, we conducted sensitivity analysis exclusively on the 90 participants who received dapagliflozin. A two-sided *p* < 0.05 represented the statistically significant. Figures were produced by GraphPad Prism version 8.0 (GraphPad Software, San Diego, CA, United States).

## 3. Results

### 3.1. Characteristics of study population

Among the 142 patients with IgAN and SGLT2i therapy, 93 patients who received at least three months of treatment were included in this study, and 62 patients completed six months of treatment. Three patients were treated with canagliflozin, and the remaining 90 received dapagliflozin. The baseline characteristics of the study population are summarized in Table 1. In summary, the mean age of the participants was 43 years, with 55 (59.1%) being male. At baseline, 17 (18.3%) patients had diabetes, and 81 (87.1%) patients had hypertension. The mean values of SBP and DBP were 124 ± 12 mmHg and 80 ± 9 mmHg, respectively. The average eGFR was 58 ± 22 mL/min/1.73m^2^, and the median proteinuria was 1.32 (IQR, 1.00–2.24) g/24 h. The mean level of serum albumin was 40.7 ± 4.0 g/L. The median urine sodium excretion was 162 (126–209) mmol/24 h and the initial hematuria was 12.3 (IQR, 3.5–38.2) RBC/HPF. Overall, 8 (8.6), 23 (24.7) and 6 (6.5) participants received glucocorticoid, hydroxychloroquine and mycophenolate mofetil treatment before and during SGLT2i administration, respectively.

**Table 1:** Baseline characteristics of patients.

Characteristics	Baseline (*n* = 93)
Age, yr., mean ± SD	43 ± 11
Male, *n* (%)	55 (59.1)
Hypertension, *n* (%)	81 (87.1)
Diabetes, *n* (%)	17 (18.3)
Initial proteinuria, g/24 h, median, IQR	1.32 (1.00 to 2.24)
Albumin, g/L	40.7 ± 4.0
eGFR, ml/min/1.73m^2^, mean ± SD	58 ± 22
SBP, mmHg, mean ± SD	124 ± 12
DBP, mmHg, mean ± SD	80 ± 9
Urine sodium excretion, median, IQR	162 (126 to 209)
Hematuria, RBC/HPF	12.3 (3.5 to 38.2)
Oxford classification, *n* (%)^a^	
M1	47 (56.0)
E1	24 (28.6)
S1	65 (77.4)
T1/T2	22 (26.2) / 8 (9.5)
C1/C2	488 (57.1) / 7 (8.3)
Medication, *n* (%)	
Glucocorticoid	8 (8.6)
Hydroxychloroquine	23 (24.7)
Mycophenolate mofetil	6 (6.5)
Other cytotoxic drugs^b^	6 (6.5)

a The Oxford classification was developed by the Working Group of the International IgA Nephropathy Network and the Renal Pathology Society. Oxford scores of 9 participants were unavailable because each of them had fewer than eight glomeruli.

b Other cytotoxic drugs include cyclophosphamide, cyclosporin A, FK506 and leflunomide; SD, standard deviation; IQR, interquartile range; eGFR, estimated glomerular filtration rate; SBP, systolic blood pressure; DBP, diastolic blood pressure; RBC, red blood cell; HPF, high-power field.

### 3.2. SGLT2i Reduces proteinuria burden in IgAN patients

**Figure 2:**
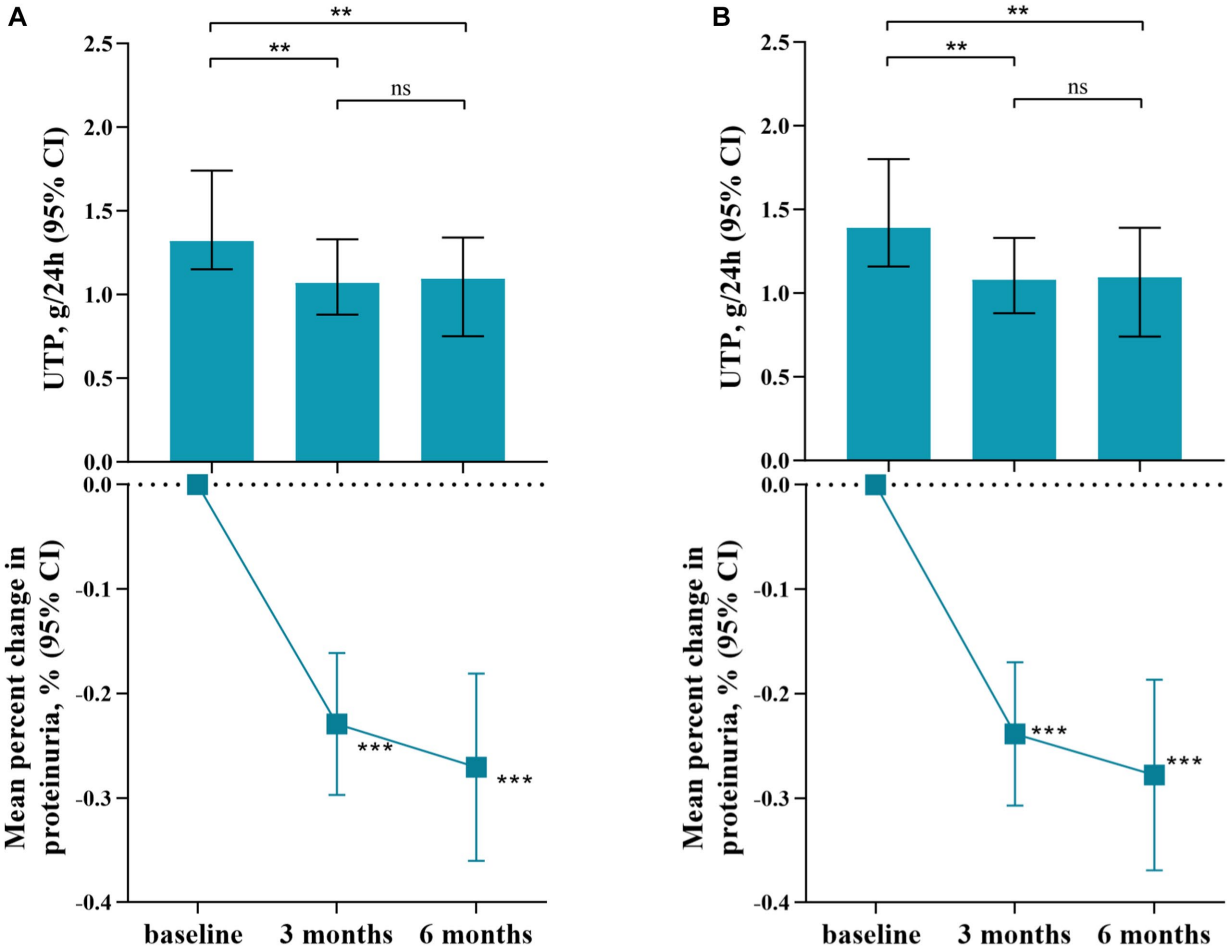
Reduction of Proteinuria Following Treatment with SGLT2 Inhibitors. **(A)**. SGLT2i group, including dapagliflozin and canagliflozin; **(B)**. Dapagliflozin group; **p value: <0.01; ***p value: <0.001. UTP: urinary total protein; CI: confidence interval.

As shown in Figure 2, therapy with SGLT2 inhibitors (SGLT2i) in IgA nephropathy (IgAN) led to a consistent reduction in proteinuria. After three months of therapy, a significant reduction in proteinuria of 22.9% [95% confidence interval (CI), 16.1 to 29.7%, *p* < 0.001] was observed compared to baseline. Similarly, at six months after therapy, the reduction in proteinuria reached 27.1% (95% CI, 18.1 to 36.0%, p < 0.001) when compared to baseline. Comparison of clinical characteristics before and after SGLT2i treatment for three months was summarized in Table 2. The median proteinuria significantly decreased from 1.32 (IQR, 1.00–2.24) g/24 h to 1.07 (IQR, 0.65–1.68) g/24 h (*p* < 0.001). Additionally, there was an increase in serum albumin levels from 40.7 ± 4.0 to 41.4 ± 4.1 g/L (*p* = 0.001). Hematuria, measured as 12.3 (IQR, 3.5–38.2) RBC/HPF before SGLT2i therapy, decreased to 8.9 (IQR, 2.6–28.8) RBC/HPF after treatment (p = 0.001). Besides, urine sodium excretion did not show a significant increase after three months of SGLT2i treatment (*p* = 0.119). Likewise, changes in clinical characteristics after six months of SGLT2i treatment were described in Table 3. As mentioned above, 62 patients underwent six months of SGLT2i therapy. The median proteinuria levels at baseline, three months, and six months after SGLT2i treatment were 1.39 (IQR, 0.99–2.35) g/24 h, 1.08 (IQR, 0.71–1.78) g/24 h, and 1.09 (IQR, 0.60–1.83) g/24 h, respectively (*p* < 0.001), indicating a significant decrease. There was an overall increase in serum albumin levels (*p* = 0.017), with values of 40.2 ± 4.4 g/L at baseline, 41.0 ± 4.0 g/L at three months, and 41.0 ± 4.1 g/L at six months after therapy. Moreover, there was a significant decrease in hematuria after six months of SGLT2i treatment (p = 0.01). After excluding individuals who were undergoing canagliflozin treatment (n = 3), there was no alteration observed in the proteinuria-reducing impact within the dapagliflozin group (n = 90): the baseline proteinuria level was 1.39 g/24 h (IQR, 1.01–2.26), which decreased to 1.08 g/24 h (IQR, 0.65–1.72) at three months and 1.10 g/24 h (IQR, 0.59–1.85) at six months (*p* < 0.001).

**Table 2:** Comparison of clinical characteristics before and after sodium-glucose cotransporter 2 inhibitors treatment for three months.

Characteristics	Baseline (*n* = 93)	Follow-up (*n* = 93)	*p* value
Initial proteinuria, g/24 h median, IQR	1.32 (1.00 to 2.24)	1.07 (0.65–1.68)	<0.001
Albumin, g/L	40.7 ± 4.0	41.4 ± 4.1	0.001
eGFR, ml/min/1.73m^2^ mean ± SD	58 ± 22	56 ± 21	0.008
SBP, mmHg, mean ± SD	124 ± 12	124 ± 13	0.947
DBP, mmHg, mean ± SD	80 ± 9	79 ± 10	0.326
Urine sodium excretion median, IQR	162 (126 to 209)	163 (126 to 195)	0.119
Hematuria, RBC/HPF	12.3 (3.5 to 38.2)	8.9 (2.6 to 28.8)	0.001

SD, standard deviation; IQR, interquartile range; eGFR, estimated glomerular filtration rate; SBP, systolic blood pressure; DBP, diastolic blood pressure; RBC, red blood cell; HPF, high-power field.

**Table 3:** The change in clinical examination after sodium-glucose cotransporter 2 inhibitors treatment for six months.

Characteristics	Baseline (*n* = 62)	Follow-up 1^a^ (*n* = 62)	Follow-up 2^b^ (*n* = 62)	*p* value
Initial proteinuria, g/24 h median, IQR	1.39 (0.99 to 2.35)	1.08 (0.71–1.78)	1.09 (0.60–1.83)	<0.001
Albumin, g/L, mean ± SD	40.2 ± 4.4	41.0 ± 4.0	41.0 ± 4.1	0.017
eGFR, ml/min/1.73m^2^ mean ± SD	58 ± 23	56 ± 23	55 ± 23	0.012
SBP, mmHg, mean ± SD	125 ± 12	126 ± 12	123 ± 10	0.252
DBP, mmHg, mean ± SD	80 ± 10	80 ± 9	77 ± 9	0.071
Urine sodium excretion median, IQR	160 (120 to 197)	154 (119 to 222)	158 (127 to 212)	0.042
Hematuria, RBC/μL	13.1 (4.0 to 62.2)	10.0 (3.8 to 34.7)	9.5 (1.5 to 34.6)	0.01

a Follow-up at 3 months after SGLT2i treatment.

b Follow-up at 6 months after SGLT2i treatment; SD, standard deviation; IQR, interquartile range; eGFR, estimated glomerular filtration rate; SBP, systolic blood pressure; DBP, diastolic blood pressure; RBC, red blood cell; HPF, high-power field.

**Figure 3:**
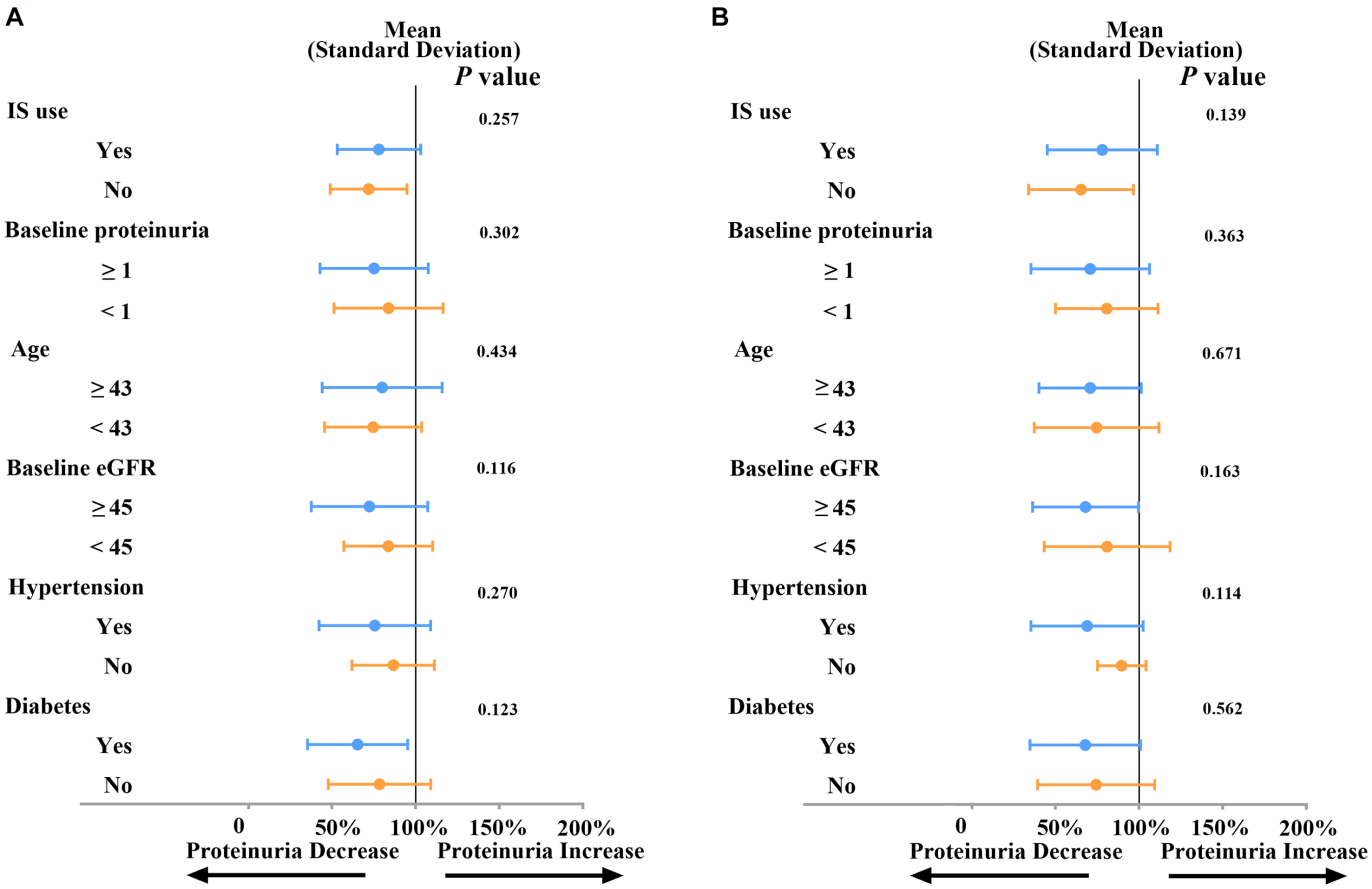
Subgroup analysis of SGLT2i on proteinuria reduction at three months **(A)** or at six months **(B)**. Proteinuria: g/L; Age: years; eGFR: ml/min/1.73m^2^; IS: immunosuppressive agents; eGFR: estimated glomerular filtration rate.

As depicted in Figure 3, the subgroup analysis revealed that the impact of SGLT2i on the reduction of proteinuria at both three months (Figure 3A) and six months (Figure 3B) did not exhibit significant differences across various baseline factors. These factors include baseline eGFR (≥45 mL/min/1.73 m^2^ or < 45 mL/min/1.73 m^2^, *p* = 0.116 for three months and *p* = 0.163 for six months), proteinuria (≥1 g/L or < 1 g/L, *p* = 0.302 for three months and *p* = 0.363 for six months), age (≥43 or < 43, *p* = 0.434 for three months and *p* = 0.671 for six months), the use of immunosuppressive agents (*p* = 0.257 for three months and *p* = 0.139 for six months), or status of diabetes (*p* = 0.123 for three months and *p* = 0.562 for six months) and hypertension (*p* = 0.27 for three months and *p* = 0.114 for six months).

### 3.3. Effect of SGLT2i treatment on estimated GFR and blood pressure

**Figure 4:**
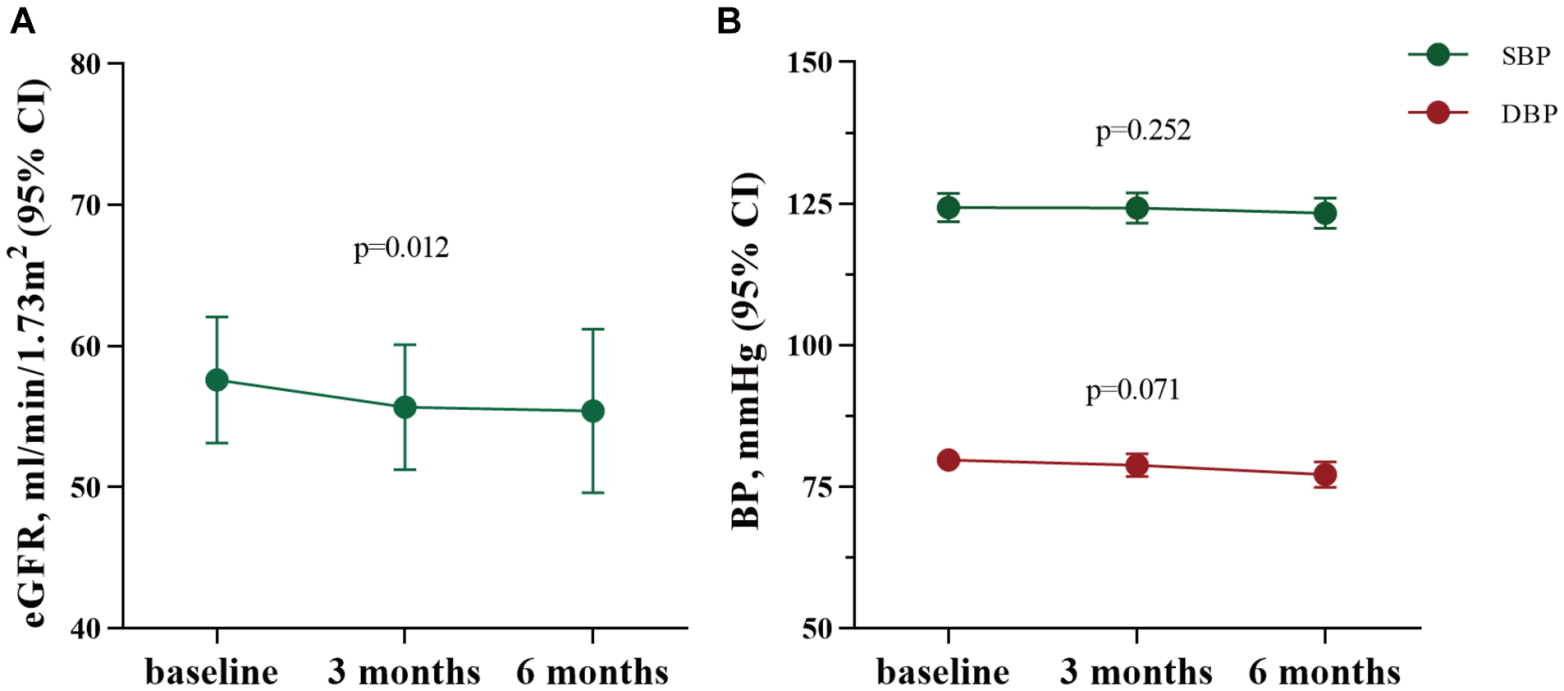
The changes of eGFR, SBP and DBP after SGLT2i treatment for three months (*n* = 93) and six months (*n* = 62). eGFR: estimated glomerular filtration rate; CI, confidence interval; BP, blood pressure; SBP, systolic blood pressure; DBP, diastolic blood pressure.

During the therapy with SGLT2 inhibitors (SGLT2i), a mild decrease in estimated glomerular filtration rate (eGFR) was observed. The eGFR decreased from 58 ± 22 mL/min/1.73m^2^ before therapy to 56 ± 21 mL/min/1.73m^2^ three months after therapy (*p* = 0.008), and from 58 ± 23 mL/min/1.73m^2^ to 55 ± 23 mL/min/1.73m^2^ after six months SGLT2i treatment (*p* = 0.012). While there was no significant difference at three and six months (as shown in Figure 4A). Moreover, a slight positive correlation (R = 0.24, *p* = 0.02) between the decrease in eGFR and proteinuria following treatment with SGTL2i was observed. The changes of SBP and DBP were not statistically significant. After three months of SGLT2i therapy, SBP showed little change from 124 ± 12 mmHg to 124 ± 13 mmHg (*p* = 0.947), and DBP exhibited a mild decline from 80 ± 9 mmHg to 79 ± 10 mmHg (*p* = 0.326). Similarly, after six months of SGLT2i administration, SBP changed from 125 ± 12 mmHg to 123 ± 10 mmHg (*p* = 0.252), and DBP presented a decrease from 80 ± 10 mmHg to 77 ± 9 mmHg (*p* = 0.071) (as shown in Figure 4B).

## 4. Discussion

In the present study of 93 patients with IgAN, we found that treatment with SGLT2 inhibitors (SGLT2i) for three and six months led to a notable reduction in proteinuria. Specifically, we observed a significant proteinuria reduction of 22.9 and 27.1% relative to the baseline after three and six months of SGLT2i treatment, respectively. Importantly, this effect was independent of various factors including age, baseline proteinuria, eGFR level, hypertension, immunosuppressive agent therapy, or the presence of comorbid diabetes. These findings highlight the potential of SGLT2i as a promising therapeutic approach for reducing proteinuria in patients with IgAN.

Still, supportive therapy remained the cornerstone of IgAN management. The TESTING study revealed that IgAN patients who did not achieve proteinuria remission with supportive therapy had a significantly high risk of kidney failure, with an annual end-stage kidney disease (ESKD) rate as high as 7.8% (2). While the addition of steroids further reduced proteinuria and the risk of kidney failure, it also led to an increase in the incidence of serious adverse events. In a recent subgroup analysis of the DAPA-CKD trial, which focused on 270 patients with IgAN, dapagliflozin demonstrated a mean percentage difference of −35.0% in the urinary albumin-to-creatinine ratio (UACR) compared to placebo in the fourth month (5, 9). Notably, the proteinuria-reducing effect of dapagliflozin was sustained throughout the follow-up period. The effect size of SGLT2i observed in this cohort study aligned with the findings of the DAPA-CKD trial, suggesting that SGLT2i could be a viable option for regular supportive therapy in IgAN.

Previously, most of the research related to SGLT2i focused on evaluating their renal protective effect exclusively in the diabetic population. A *post hoc* study conducted during the CREDENCE trial shed light on this matter. The study revealed that canagliflozin, when compared to the placebo group, exhibited a significant reduction of 31% in UACR levels after 26 weeks of treatment in patients with diabetic kidney disease and macroproteinuria (UACR >300 mg/g) (13). Similarly, the investigators of the CANVAS trial specifically selected diabetes patients with micro or macroalbuminuria. They observed that canagliflozin led to a substantial decrease of 40.4% in UACR burden among this population after 1 year of follow-up (14). The DAPA-CKD study stands as the first trial to include approximately one-third of patients with non-diabetic kidney disease, aiming to assess the therapeutic effect of SGLT2 inhibitors. The results from this study indicated that dapagliflozin provided comparable kidney protection for both patients with diabetic and non-diabetic kidney disease (15).

The SGLT2i functions within the proximal tubule by inhibiting the reabsorption of sodium and glucose through SGLT2. As a result, there is an increased delivery of sodium, chloride, and water to the macula densa, leading to tubuloglomerular feedback. This feedback mechanism induces vasodilation in the glomerular afferent arterioles, reducing intraglomerular perfusion pressure and overall filtration. Consequently, this effect contributes to a decrease in urinary protein excretion, potentially making SGLT2i a viable treatment option for glomerular diseases in non-diabetic patients. However, findings from the DIAMOND study demonstrated that dapagliflozin, a specific SGLT2i, did not yield any additional reduction in proteinuria levels among patients with non-diabetic chronic kidney disease who were already receiving RAAS blockers (16). It is worth noting that the patients in the DIAMOND study were administered a dosage of 10 mg/day of dapagliflozin for a duration of six weeks. This relatively short treatment period may not have been sufficient to observe a therapeutic effect.

Until now, there have been no published trials providing powerful evidence for the efficacy of SGLT2i in treating nondiabetic kidney disease. However, randomized controlled trials are currently underway, specifically focusing on the potential of SGLT2i to decrease proteinuria in nondiabetic patients. One such trial is the ADAPT trial (NCT04794517), which has enrolled patients with stage IV chronic kidney disease (CKD) unrelated to diabetes. The participants in the ADAPT trial have been randomly divided into two groups: the interventional group receiving a daily dose of 10 mg dapagliflozin, and the placebo group. The trial has a follow-up period of six weeks, during which the aim is to evaluate the short-term impact of dapagliflozin on reducing proteinuria in this particular population. However, larger-scale trials with longer follow-up focusing on glomerular disease or patients with nondiabetic proteinuria are still required in the future to gather more comprehensive data.

Our research is subject to several limitations that should be considered. Firstly, we employed a prospective uncontrolled design, which introduces the possibility of selection bias and may impact the interpretation of the results. Secondly, the number of participants in our study was limited, and all data were collected through self-reporting. Therefore, it is important to conduct additional trials with a control group and a larger sample size to validate our findings. Notably, ongoing trial NCT04662723 may provide further insights into this matter. Thirdly, the follow-up duration in our study was relatively short. Therefore, it is necessary to conduct further investigations to explore the long-term effects of SGLT2i on proteinuria in patients with IgAN.

In conclusion, treatment with SGLT2i has consistently demonstrated an antiproteinuric effect in patients with IgAN across various clinical settings. This effect remains significant irrespective of factors such as age, concomitant immunosuppressive agent therapy, baseline proteinuria/eGFR level, and blood pressure/glucose status. Further studies are necessary to investigate the long-term renal protective effects of SGLT2i in patients with IgAN.

## Data availability statement

The raw data supporting the conclusions of this article will be made available by the authors, without undue reservation.

## Ethics statement

The studies involving humans were approved by Peking University First Hospital ethics committee. The studies were conducted in accordance with the local legislation and institutional requirements. Written informed consent for participation was not required from the participants or the participants’ legal guardians/next of kin in accordance with the national legislation and institutional requirements.

## Author contributions

YD designed the research, analyzed the data, and wrote the manuscript. SS, LL, XZ, and HZ provided supervision. JL provided critical revisions to the manuscript. All authors contributed to the article and approved the submitted version.

## Funding

This work was supported by the National Natural Science Foundation of China (81925006 and 82070733), the Capital of Clinical Characteristics, the Applied Research Fund (Z161100000516005), Grants from the Science and Technology Project of Beijing, China (D18110000011803), and CAMS Innovation Fund for Medical Sciences (2019-1 2 M-5-046).

## Conflict of interest

The authors declare that the research was conducted in the absence of any commercial or financial relationships that could be construed as a potential conflict of interest.

## Publisher’s note

All claims expressed in this article are solely those of the authors and do not necessarily represent those of their affiliated organizations, or those of the publisher, the editors and the reviewers. Any product that may be evaluated in this article, or claim that may be made by its manufacturer, is not guaranteed or endorsed by the publisher.
